# Effects of Vitamin E-Stabilized Ultra High Molecular Weight Polyethylene on Oxidative Stress Response and Osteoimmunological Response in Human Osteoblast

**DOI:** 10.3389/fendo.2019.00203

**Published:** 2019-04-03

**Authors:** Luca Massaccesi, Vincenza Ragone, Nadia Papini, Giancarlo Goi, Massimiliano Marco Corsi Romanelli, Emanuela Galliera

**Affiliations:** ^1^Department of Biomedical Sciences for Health, Università degli Studi di Milano, Milan, Italy; ^2^Research and Develpoment Department, Permedica S.p.A, Merate, Italy; ^3^Department of Medical Biotechnology and Traslational Medicine, Università degli Studi di Milano, Milan, Italy; ^4^Department of Biomedical, Surgical and Dental Sciences, Università degli Studi di Milano, Milan, Italy; ^5^U.O.C SMEL-1 Patologia Clinica San Donato, IRCCS Policlinico San Donato, Milan, Italy; ^6^IRCCS Istituto Ortopedico Galeazzi, Milan, Italy

**Keywords:** vitamin E, high-molecular-weight polyethylene (HMWPE), oxidative stress, osteoblasts, proteins O-GlcNAcylation, osteoimmunological markers

## Abstract

High Crosslink process was introduced in the development of joint prosthetic devices, in order to decrease the wear rate of ultrahigh molecular weight polyethylene (UHMWPE), but it also triggers the formation of free radicals and oxidative stress, which affects the physiological bone remodeling, leading to osteolysis. Vitamin E stabilization of UHMWPE was proposed to provide oxidation resistance without affecting mechanical properties and fatigue strength. The aim of this study is to evaluate the antioxidant effect of vitamin E added to UHMWPE on oxidative stress induced osteolysis, focusing in particular on the oxidative stress response in correlation with the production of osteoimmunological markers, Sclerostin and DKK-1, and the RANKL/OPG ratio compared to conventional UHMWPE wear debris. Human osteoblastic cell line SaOS2 were incubated for 96 h with wear particles derived from crosslinked and not crosslinked Vitamin E-stabilized, UHMWPE without Vitamin E, and growth medium as control. Cellular response to oxidative stress, compared to not treat cells, was evaluated in terms of proteins O-GlcNAcylation, cellular levels of OGA, and OGT proteins by immunoblotting. O-GlcNAcylation and its positive regulator OGT levels are increased in the presence of Vitamin E blended UHMWPE, in particular with not crosslinked Vit E stabilized UHMWPE. Conversely, the negative regulator OGA increased in the presence of UHMWPE not blended with Vitamin E. Vitamin E-stabilized UHMWPE induced a decrease of RANKL/OPG ratio compared to UHMWPE without Vitamin E, and the same effect was observed for Sclerostin, while DKK-1 was not significantly affected. In conclusion, Vitamin E stabilization of UHMWPE increased osteoblast response to oxidative stress, inducing a cellular mechanism aimed at cell survival. Vitamin E antioxidant effect influences the secretion of osteoimmunological factors, shifting the bone turnover balance toward bone protection stimuli. This suggests that Vitamin E-Stabilization of UHMWPE could contribute to reduction of oxidation-induced osteolysis and the consequent loosening of the prosthetic devices, therefore improving the longevity of total joint replacements.

## Introduction

One of the main problems in total hip arthroplasty is osteolysis triggered by ultrahigh molecular weight polyethylene (UHMWPE) wear particles (1) and different strategies have been developed to improve the oxidation and wear resistance.

High crosslink process was developed in order to decrease the wear rate of UHMWPE (2), but it also triggers the formation of free radicals (3), leading to oxidative degradation of the material through a cascade reaction with oxygen (4). In order to reduce oxidation, Vitamin E was introduced in UHMWPE stabilization, to provide oxidation resistance without affecting mechanical and fatigue strength of the material (5, 6). Vitamin E in the most abundant and effective antioxidant in the body, able to react with free radicals in cell membrane and protect polyunsaturated fatty acids from degradation due to oxidation (7). Polyethylene has a lipid—like molecular structure and its oxidation follows a similar mechanism of oxidation of lipids *in vivo* (8).

The physiological intracellular redox state is maintained in equilibrium by the balance of antioxidants and reactive oxygen species (ROS)- producing enzyme. Oxidative stress occurs when the overproduction of ROS is not balanced by an adequate level of antioxidants (9). The redox state affects the physiological process of bone remodeling (9). Indeed, changes in the ROS/antioxidant balance are involved in the pathogenesis of bone loss. In particular, oxidative stress activates the differentiation of osteoclasts from their precursors, while inducing osteoblasts apoptosis, thus shifting the balance toward osteoclastogenesis and bone resorption (9). High levels of ROS reduce osteoblast differentiation and activity, therefore reducing mineralization and bone mass (10, 11). On the contrary, antioxidant may contribute to osteoblasts differentiation and activity, promoting bone formation (9, 11).

Moreover, oxidative stress promotes the inflammatory response and directly interferes with the osteoimmunological regulation of bone remodeling, based on the action of the RANKL/RANK/OPG system. The receptor activator of NF-κB (nuclear factor-κB) ligand (RANKL) is a key factor stimulating the differentiation and activation of osteoclasts, and therefore, is essential for bone remodeling. The binding of RANKL to its receptor RANK leads to osteoclasts differentiation, while the decoy receptor Osteoprotegerin (OPG) counteracts this effect by binding and blocking RANKL. ROS act as specific secondary messengers in signaling pathways involved in RANKL-induced osteoclast differentiation (12). The expression of RANKL and OPG is sensitive to oxidative status that reduces OPG expression and induces RANKL expression, thus shifting the balance toward bone loss. An excess of oxidative stress also induces apoptosis of osteocytes, resulting in the reduction OPG production and increase of Sclerostin and DKK-1, two inhibitors of the WNT pathway involved in the osteoimmunological regulation of bone remodeling (13, 14). The expression of WNT pathway inhibitors seems to be induced by inflammatory mediators and aging (15), both conditions characterized by an increase in oxidative stress.

In this context, knowing that oxidative stress plays an important role in bone remodeling disorders, it's extremely important to study the effect of antioxidant agent (Vitamin E) added to UHMWPE that may reduce oxidative stress, thus modulating the inflammatory process and the regulation of bone remodeling by osteoimmunological mediators, eventually protecting from periprosthetic bone loss. Several studies in the recent years evaluated the clinical advantages of Vitamin E added to UHMWPE (16–20), but there is little if no evidence focusing in particular on the protective role of Vitamin E from oxidative stress in correlation with inflammation and the consequent periprosthetic bone loss.

One of the main mechanisms of cellular oxidative stress response is the modification of intracellular proteins by monosaccharides of O-linked β-N- acetylglucosammine, known as O-GlcNAcylation (21). This is a post-translational modification of nuclear and cytoplasmic proteins, which consists in the attachment of a single N-acetylglucosamine (O-GlcNAc) to serine and threonine residues of a protein (22).Two enzymes regulate this process: O-GlcNAc transferase (OGT) that catalyzes the addition of O-GlcNAc to the hydroxyl group of serine or threonine residues of a protein, and O-GlcNAcase (OGA) that removes O-GlcNAc from proteins (23, 24). Levels of O-GlcNAc are induced in response to stress, in order to prevent apoptosis and promote cell survival mechanism, and are considered a target of mammalian stress response (25). The protein O-GlcNAcylation, OGA and OGT levels are therefore considered markers of cellular response to oxidative stress.

The aim of this study is to evaluate the antioxidant effect of vitamin E added to UHMWPE and the association with the main osteoimmunological biomarkers (RANKL/RANK/OPG), and WNT pathway inhibitors, in order to better understand how the antioxidant effect of Vitamin E can prevent periprosthetic inflammation and the consequent o and prosthetic loosening.

## Methods

### UHMWPE Particles Generation

UHMWPE wear particles were generated as previously described in Galliera et al.(26) by four different UHMWPE articular inserts (raw material GUR 1020): Material (1) moderately cross-linked vitamin E-blended UHMWPE (60 kGy electron-beam irradiated) (vitamin E concentration 0.1 wt%), EtO sterilized (*Vital-XE*®*, Permedica S.p.A*.); Material (2) standard UHMWPE (without vitamin E and not cross-linked), EtO sterilized *(Permedica S.p.A);* Material (3) vitamin E-blended UHMWPE (vitamin E concentration 0.1 wt%) not cross-linked, EtO sterilized (*Vital-E*®*, Permedica S.p.A*.).

All UHMWPE wear particles were generated as described previously (26). Briefly, the articular inserts were rubbed for 10 days at 230 rpm against ceramic ball heads using a combined drilling and tapping machine (IM company, Italy), applying a load of 1,000 N. The resulting UHMWPE particles were released directly into a closed sterile recipient containing 500 mL of ultrapure water with 0.2% sodium azide (antibacterial additive).

### Cell Culture and Treatment With Wear Particles

SAoS2 cells, a permanent line of human osteoblast-like cells, were obtained from a partner institute and grown in RPMI 1,640 (Invitrogen, Germany) with L-glutamine, 10% fetal bovine serum (FBS), 100 U/mL penicillin and 100 μg/mL streptomycin (GIBCO, USA). Cells were cultured in 5% CO_2_ at 37°C in 12-well culture plates (Corning, USA).

Treatment with HUMWPE wear particles was performed as previously described (26). Briefly, after sterilization by UV irradiation overnight, the three types of wear particle (described above) diluted in growth media at a concentration of 1:1 or pure media (as control) were added to the cell culture. After 96 h of incubation, the supernatants of each well were collected and stocked at −20°C for ELISA assays, while cells were washed with PBS twice and then lysed for 15 min at 4°C in lysis buffer (25 mM Tris-HCl pH 7.4, 150 mM NaCl, 5 mM EDTA, 20 mM NaF, 1 mM Na3VO4, 0.5% v/v NP40, 10 mg/ml leupeptin, 10 mg/ml aprotinin, 1 mg/ml pepstatin A). Insoluble material was removed by centrifugation at 13,000 g for 10 min, supernatants were collected and assayed for protein concentration with Coomassie Protein Assay (Pierce). Then samples were analyzed by immunoblotting.

### Evaluation of Oxidative Stress Parameters: Immunoblotting and Densitometry Analysis

Forty micrograms of cell proteins were separated by SDS electrophoresis under denaturating conditions using 6–10% polyacrylamide gels. SDS-PAGE gels were electrophoretically transferred on PVDF membrane in Tris-glicine buffer, using the Mini Transblot System (Bio-Rad Laboratories, Richmond, VA). O-GlcNAc levels were measured by anti-b-O-linked N-Acetylglucosamine (OGlcNAc) CTD 110.6, an antibody that specifically recognizes endogenous levels of O-GlcNAc, linked to both serine and threonine residues of proteins, 1:1000 dilution (Cell Signaling). Other primary antibodies were used as follows: anti-OGA 1:3000 dilution (Sigma-Aldrich), anti-OGT 1:500 dilution (Sigma–Aldrich), and anti-Histone H3 1:2000 dilution (Cell Signaling).

Each membrane was washed three times for 10 min and then incubated with the appropriate secondary antibody conjugated with horseradish peroxidase (Santa Cruz Biotechnology) for 1 h. For the immunological detection of proteins, MINI HD 9 System (Uvitec Limited, Cambridge UK) was used. Band density was quantified Quantity One Software (Bio-Rad Laboratories).

### Evaluation of Oxidative Stress Parameters: ROS Generation Assay

ROS production was tested by OxiSelect™ *In vitro* ROS/RNS Assay kit [Green Fluorescence (Cell Biolabs)], according to manufacturer's protocol. Briefly, the OxiSelect™ *In vitro* ROS/RNS Assay Kit is an *in vitro* assay for measuring total ROS/RNS free radical activity. Unknown ROS or RNS samples or standards are added to the wells with a catalyst that helps accelerate the oxidative reaction. After a brief incubation, the prepared DCFH probe is added to all wells and the oxidation reaction is allowed to proceed. Samples are measured fluorometrically against a hydrogen peroxide or DCF standard. The assay is performed in a 96-well fluorescence plate format that can be read on a standard fluorescence plate reader. The free radical content in unknown samples is determined by comparison with the predetermined DCF or hydrogen peroxide standard curve.

### Evaluation of Osteoimmunological Biomarkers: ELISA Assay

The osteoimmunological biomarkers were evaluated by ELISA assay in SaOS2 supernatant. In particular, RANKL was measured using an ELISA Duo Set assay (R&D System, Minneapolis, MN, USA), while DKK-1, OPG, and Sclerostin were measured by ELISA Quantikine colorimetric sandwich assays (R&D System, Minneapolis, MN, USA), according to the manufacturer's protocols.

RANKL: CV intra assay 8.01% and inter assay 6.2%; OPG: CV intra assay 7.3% and inter assay 6.9%; DKK-1: CV intra assay 2.7 % and inter assay 5.4 %, SOST: CV intra assay 2.1 %, and inter assay 8.2%.

### Evaluation of Cell Vitality

Cell viability was assessed quantitatively using the resulting Alamar Blue® test, a non-toxic test for cells as it exploits the reducing power of living cells by measuring their metabolic activity quantitatively, which makes it possible to analyze, in different timing, the same proliferating cell population. This test was also used as an indirect index of cell proliferation. The assay exploits the mitochondrial activity of viable cells capable of maintaining an environment of reducing inside the cell. Resazurin, the active component of Alamar Blu, is a compound able to cross the membranes, and once it enters the cell, it gets reduced in Resorufin and its color turns from blue to red. The reduction was measured by a spectrophotometer at 560 and 600 nm (Glo Max, Promega). The results obtained by both the readings were analyzed following the indications provided by the assay protocol by calculating the percentage of cell viability as the difference in the reduction between the treated samples and the samples control (medium + Alamar Blue®).

### Evaluation of Mineralization

The cells were seeded in 6-well plates at a density of 0.8 × 10^5^ cells/well and stabilized for 24 h. To induce osteoblast differentiation, Osteogenic medium (Promocell), containing 50 μg/ml L-ascorbic acid and 10 mM β-glycerophosphate, was added to culture for 11 days. The culture medium was changed every 3–4 days. Then, the cells were fixed in 10% formalin for 10 min and stained with the 40 mM Alizarin Red-S (pH 4.2; Sigma-Aldrich; Merck KGaA) for 15 min, all at RT. For quantification of Alizarin red S, 500 μl citrate solution containing 20% methanol and 10% acetic acid was added for 20 min at RT, and the absorbance of supernatants was measured at 570 nm using a GloMAx Fluorescence Reader (Promega). The plates were observed under the Leica Microscope DML B2/11888111 equipped with Leica camera DFC450 at × 100 magnification.

### Statistical Analysis

Statistical analysis was performed with dedicated statistical software (GraphPad Prism 7); normality of distribution of the groups was verified by KS normality for all the parameters evaluated. Statistical analysis was performed with one-way ANOVA, considering *p* < 0.05 as significant and *p* < 0.001 as highly significant. Data are expressed as the mean ± standard deviation (SD).

## Results

### Oxidative Stress Response

**Figure 1:**
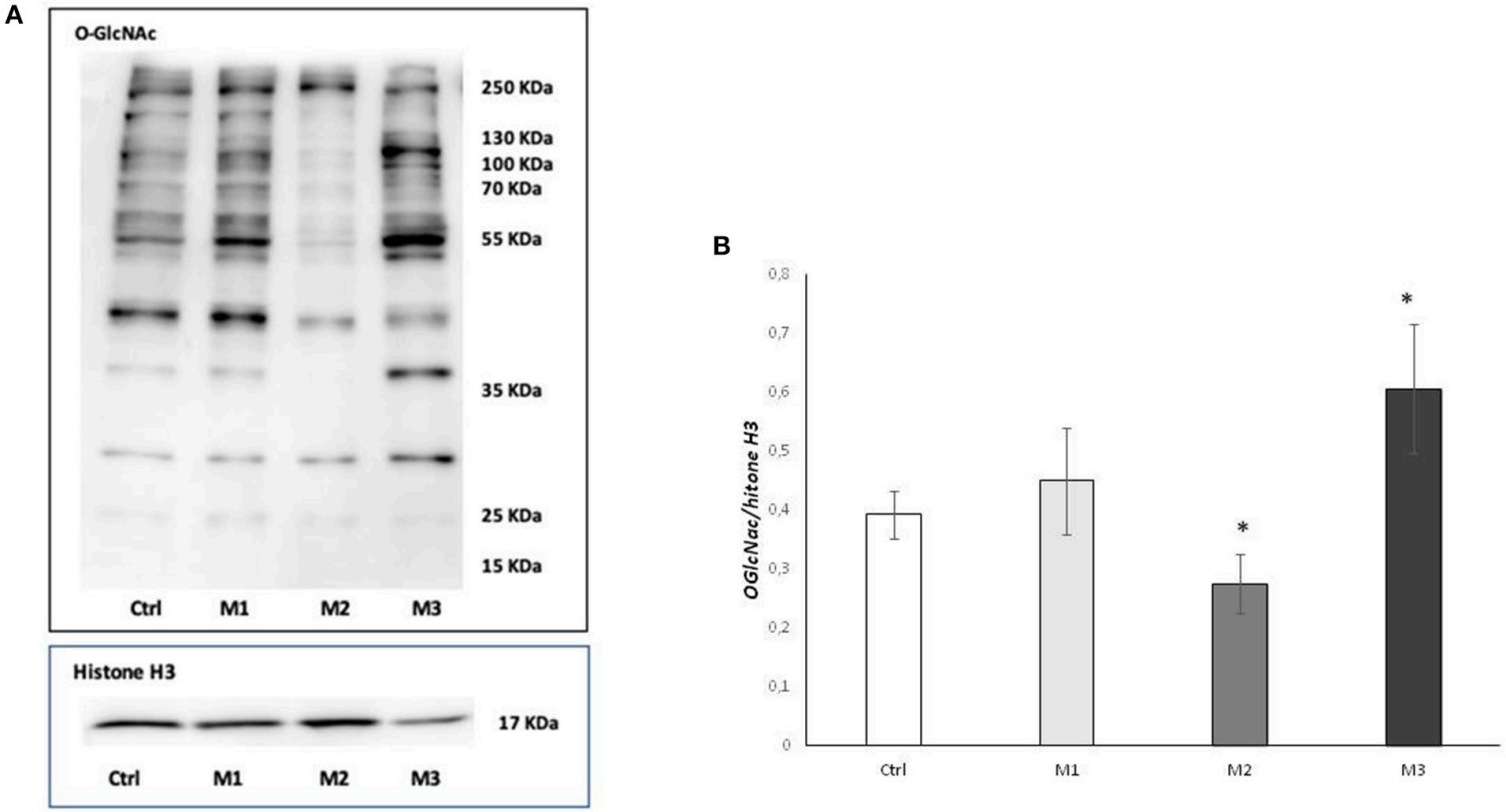
Total O-GlcNAcylations after exposure to M1, M2, and M3 wear debris. **(A)** O-GlcNAcylations of cell proteins examined by western blotting. The western blot image is representative of three experiments. **(B)** Densitometric analysis of proteins expression was performed using Histone H3 as loading control. *P < 0.05 vs. Ctrl. Materials M1, M2, M3 are described in the Methods section, as follows: Material M1 a moderately cross-linked vitamin E-blended UHMWPE, EtO sterilized; Material M2 standard UHMWPE (without vitamin E and not cross-linked), EtO sterilized; Material M3 vitamin E-blended UHMWPE not cross-linked, EtO sterilized. Cnotrol (white bar) Material M1 (light gray bar), material M2 (medium gray bar), material M3 (dark gray bar).

To investigate the effect of the of Vitamin E blended UHMWPE on O-GlcNAcylation process, we measured cellular O-GlcNAC levels by western blot using CTD110.6 antibody. As shown in Figure 1, Panel A, O-GlcNAcylation levels increased in the presence of Vitamin E blended UHMWPE (in particular with not crosslinked Vit E blended UHMWPE) while, conversely, they fall in the absence of Vitamin E.

Densitometric analysis showed a significative (*p* < 0.005) increase in O-GlcNAC levels in the presence of not-crosslinked Vit E blended HMWPE and a significative (*p* < 0.005) decrease in Vitamin E absence (Figure 1B).

**Figure 2:**
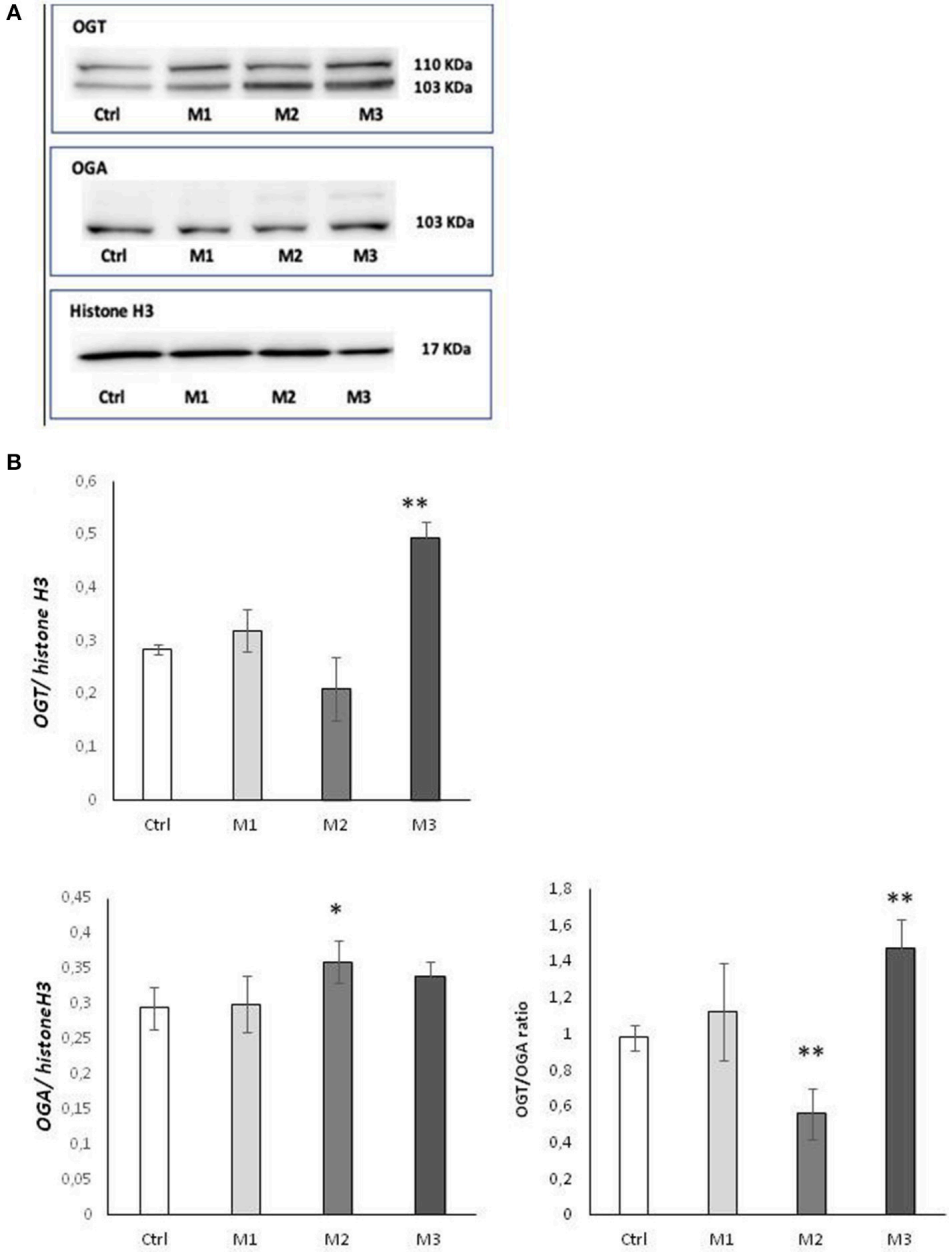
OGA and OGT levels after exposure to M1, M2, and M3 wear debris. **(A)** Cellular levels of OGA and OGT proteins examined by western blotting. The western blot image is representative of three experiments. Densitometric analysis of proteins expression was performed using Histone H3 as loading control. **(B)** OGT/OGA expression ratio. *P < 0.05 Vs. Ctrl; **P < 0.01 Vs. Ctrl. Materials M1, M2, M3 are described in the Methods section, as follows: Material M1 a moderately cross-linked vitamin E-blended UHMWPE, EtO sterilized; Material M2 standard UHMWPE (without vitamin E and not cross-linked), EtO sterilized; Material M3 vitamin E-blended UHMWPE not cross-linked, EtO sterilized. Control (white bar) Material M1 (light gray bar), material M2 (medium gray bar), material M3 (dark gray bar).

In order to determine whether the observed variation in O-GlcNAC levels was caused by an alteration of the ratio between the two O-GlcNAc cycling enzymes, we examined protein expression of OGA and OGT by western blot analysis (Figures 2A,B). As shown in Panel A, a significant increase (*p* < 0.01) of OGT protein level was found in the presence of not-crosslinked Vitamin E blended UHMWPE, whereas a significant increase (*p* < 0.05) of OGA enzyme was observed in Vitamin E absence. The OGT/OGA expression ratio shows a behavior consistent with the observed O-GlcNAC levels (Figure 2B).

ROS production was evaluated during the treatment, at 24, 48, 72, and 96 h (**Figure 4C**).The results show that at early time points (24 h), there is a significative increase of ROS induced by M1 (crosslinked HUMWPE vitamin E added), while M3 (not crosslinked V, Vitamin E added UHMWPE) display a significative decrease compared to M2 (not crosslinked V, UHMWPE without Vitamin E). This effect attenuates at 48 h, resulting in an insignificant difference between M1 and M2, maintaining a low but weakly significant level in response to M3. At 72 h, there is a little and insignificant increase in ROS in response to M1 and M3, which then turns into comparable levels, with no significative difference between the three materials at 96 h.

### Osteoimmunological Biomarkers

The secretion of osteoimmunological markers in the supernatant was measured after 96 h of incubation with material 1, 2, and 3, as described in the Method Session, and growth medium as control. The two main osteoimmunological pathways were evaluated: the RANK/RANKL/OPG, by measuring the secretion of RANKL and OPG from the osteoblast cell line SaOs2 pathway, and the Wnt pathway, by measuring the secretion of the two main Wnt inhibitors, Sclerostin (SOST), and DKK-1.

**Figure 3:**
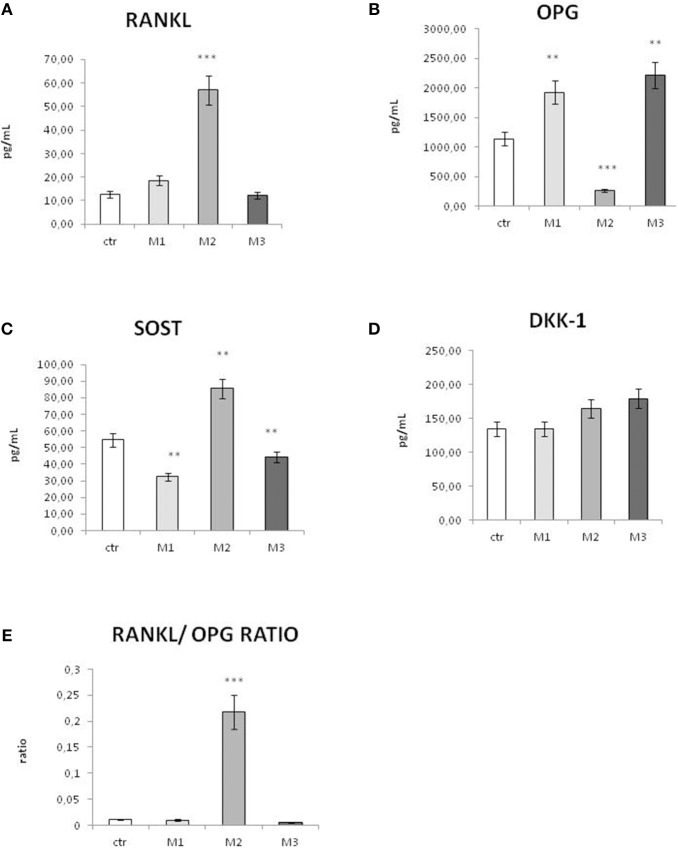
Secretion of osteoimmunological biomarkers in the cell culture supernatant after exposure to M1, M2, and M3 wear debris. Concentrations (picograms per milliliter) of RANKL **(A)**, OPG **(B)**, Sclerostin (SOST, **C)**, and DKK-1 **(D)**, RANKL/OPG ratio **(E)** in the cell culture supernatant of control (white bar), material M1 (light gray bars), material M2 (medium gray bars), material M3 (dark gray bars). Materials M1, M2, M3 are described in the Methods section, as follows: Material M1 a moderately cross-linked vitamin E-blended UHMWPE, EtO sterilized; Material M2 standard UHMWPE (without vitamin E and not cross-linked), EtO sterilized; Material M3 vitamin E-blended UHMWPE not cross-linked, EtO sterilized. Control (white bar) Material M1 (light gray bar), material M2 (medium gray bar), material M3 (dark gray bar). *p < 0.05, statistically significant; **p < 0.01; ***p < 0.005.

RANKL production (Figure 3A) displayed a insignificant increase in response to M1 material, compared to control, and it resulted comparable to control in response to M3 material. On the contrary, RANKL displayed a strong and significant increase in response to material M2. Conversely, OPG (Figure 3B) displayed a significant reduction in response to material M2, while it showed a significant increase in response to M1 and an even higher increase in response to material M3.

In order to evaluate the trend of bone remodeling regulators *in vitro*, the RANKL/OPG ratio was calculated (Figure 3E). In all the conditions, the RANKL/OPG ratio resulted largely <1. However, while in response to M1 and M3 the RANKL/OPG ratio was very low and comparable to control, in response to M2 the RANKL/OPG ratio displayed a very significant increase.

Sclerostin, a marker of bone resorption, displayed a significant increase in response to material M2, while it showed a significant decrease in response to material 1 and a little, even significant decrease in response to material M3 (Figure 3C). The secretion of the other Wnt inhibitor DKK-1 displayed no significant changes in response to material M1 compared to control (Figure 3D).

### Cell Vitality and Mineralization After Exposure to M1, M2 and M3 Wear Debris

Cell were plated at the concentration of 10^3^cells/mL, and 24 h after, when they resulted completely adherent, the incubation with wear particles started and lasted for 96 h, during the exponential growth phase. Doubling time of Saos cell is 37 h and they can be cultured in logaritimic growth for 7 days before splitting, when they reach the concentration of 3 × 10^5^cells/mL.

**Figure 4:**
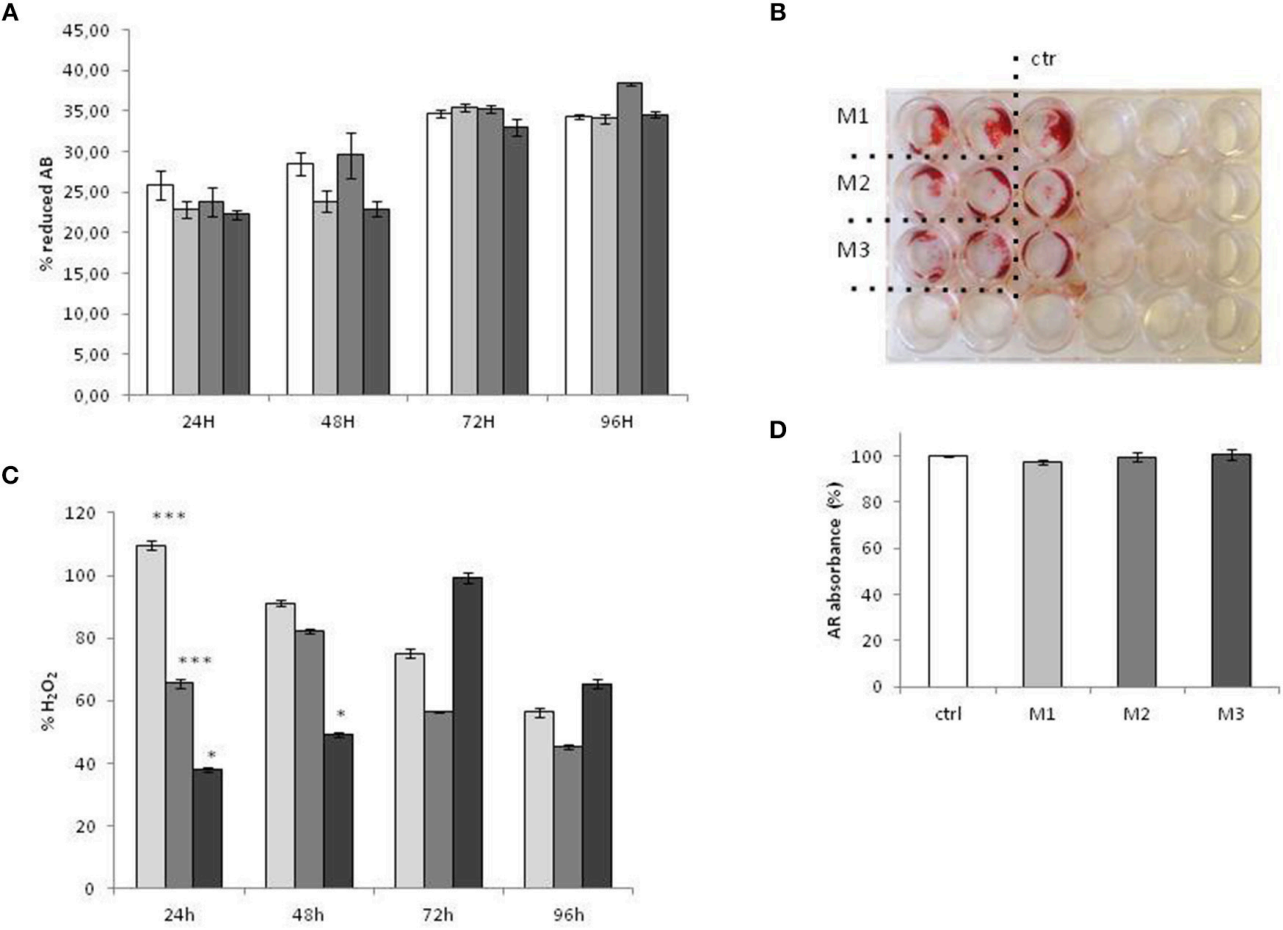
Cell Vitality, mineralization, and ROS production after exposure to M1, M2, and M3 wear debris. **(A)** vitality and the growth rate of Sa0S2 cell evaluated by Alamar blue **(A,B)**. The vitality of the cells is expressed as percentage of reduced Alamar Blue (%AB), as indicated in Alamar Blue protocol. Mineralization Assay was evaluated by alizarin Red (AR) staining. The plates were observed under the Leica Microscope DML B2/11888111 equipped with Leica camera DFC450 at × 100 magnification **(B)**. For quantification of Alizarin red S, 500 μl citrate solution containing 20% methanol and 10% acetic acid was added for 20 min at RT, and the absorbance of supernatants was measured at 570 nm using an Fluorescence Reader (GloMAx). The mineralization is expressed as percentage of Alzarin Red staining (% ARS) of treated cells vs. untreated control **(D)**. **(C)** ROS production was tested by OxiSelect™ *In vitro* ROS/RNS Assay kit. ROS production is expressed as percentage of H_2_O_2_ production vs. untreated control. Materials M1, M2, M3 are described in the Methods section, as follows: Material M1 a moderately cross-linked vitamin E-blended UHMWPE, EtO sterilized; Material M2 standard UHMWPE (without vitamin E and not cross-linked), EtO sterilized; Material M3 vitamin E-blended UHMWPE not cross-linked, EtO sterilized. Control (white bar) Material M1 (light gray bar), material M2 (medium gray bar), material M3 (dark gray bar). *p < 0.05; **p < 0.01, statistically very significant; ***p < 0.005.

Cells were equally plated for treatment with wear particles and control medium. The vitality and the growth rate of the Sa0S2 cell were evaluated by Alamar blue, and treated as well as not treated cells displayed exactly the same viability and growth rate, as shown in Figure 4A.

Mineralization assays were performed in the presence of material 1, 2, and 3, and only mineralization medium as control. As shown in Figures 4B,D. The three different kinds of material did not affect the mineralization of SaoS2.

## Discussion

Implant materials can release wear particles which may elicit adverse reactions in patients, such as local inflammatory response leading to tissue damage, which eventually results in loosening of the implant. In the case of ultra-high molecular weight polyethylene (UHMWPE), the inflammation is further boosted by the oxidation of the material, which has been recognized as a potential limiting factor for the longevity of total joint replacements (6). In order to reduce UHMWPE oxidation, chemical stabilization with Vitamin E was introduced over the past decade. Vitamin E, when added to UHMWPE, has been shown to suppress the oxidation cascade by reducing both alkyl and peroxy radicals (27). The antioxidant effect of Vitamin E has been extensively studied from the biochemical point of view (17, 28), but the comprehensive effect on cellular response to oxidative stress and the correlation with inflammatory response and bone resorption still needs to be fully elucidated. For this reason, this study aimed to evaluate the effects of Vitamin E addition to UHMWPE on both the aspects of inflammation leading to bone tissue damage: on the one hand the cellular response to oxidative stress and on the other hand the production of osteoimmunological mediators, that combine the regulation of inflammatory response to bone remodeling (23). In order to evaluate the effect of Vitamin E added, different variants of UHMWPE were evaluated. In the Vitamin E- UHMWPE production, the radiation cross linking process is required to reduce wear, but it also increases the oxidation of UHMWPE (24). For this reason, we evaluated two different types of Vitamin E-stabilized UHMWPE, one crosslinked, and one not crosslinked, compared to not crosslinked UHMWPE, without Vitamin E.

Cells and tissue respond to oxidative stress, environmental and injury by reprogramming gene expression, transcription, transduction, and post trasductional protein modification in order to stat pathways of repair and survival. In particular oxidative stress induces a dynamic O-GlcNAcylation (25, 29), promoting the glycosilation of some proteins and the deglycosilation of others, in order to prevent apoptosis and promote the cell survival mechanism (29). The dynamic O-GlcNAcylation is cycled by two enzymes, the O-GlcNAc transferase (OGT), which catalyzes the addition of O-GlcNAc residues, and the O-GlcNAcase (OGA), which removes O-GlcNAc residues. The level of O-GlcNAcylation, OGA, and OGT levels are therefore markers of cellular response to oxidative stress and were measured in the present study to evaluate the response of the osteoblastic cell line SaOs2 to wear debris from crosslinked Vitamin E—stabilized UHMWPE, not-crosslinked Vitamin E—stabilized UHMWPE and not-crosslinked UHMWPE without vitamin E, compared to not-treated cells. As shown in Figure 1, O-GlcNAcylation is significantly reduced in response to notVitamin E-stabilized UHMWPE, while they are increased in response to Vitamin E—stabilized UHMWPE, compared to controls, with a significant increase in the case of not-crosslinked Vitamin E—stabilized UHMWPE (material 3), indicating that the presence of Vitamin E increase the ability of Saos2 Cells to respond to oxidative stress due to UHMWPE. This result is also confirmed by the specific evaluation of OGA and OGT: OGT production showed the same changes observed for O-GlcNAcylation, with a significant increase in response to not-crosslinked Vitamin E—stabilized UHMWPE, a little but not significant increase in response to crosslinked Vitamin E—stabilized UHMWPE and a decrease in response to not-crosslinked UHMWPE without Vitamin E. These results are in accordance with previous reports indicating that stress induced O-GlcNAcylation is coincident with increased protein expression of OGT of OGT (29–31). OGA exert the opposite role of OGT (21), and even though less is known about the regulation of OGA levels in stresses cells, recent studies reported that increased O-GlcNAcylation levels are associated with a decrease in OGA (29, 31). Consistently with these evidences, we observed a significant increase of OGA in response to material 2 (UHMWPE without Vitamin E), in correspondence to a decrease of OGT, and no significant change in response to material 1 and 3 (UHMWPE with Vitamin E). The overall effect is more evident by evaluating OGT/OGA ratio, showing that UHMWPE without Vitamin E induced a very significant decrease in OGT/OGA ratio while not-crosslinked Vitamin E—stabilized UHMWPE induced a very significant increase in OGT/OGA ratio, shifting the balance toward increased cell survival. These results suggest that the addition of Vitamin E to UHMWPE increases the ability of SaOs2 cells to respond to oxidative stress induced by UHMWPE, while the absence of Vitamin E reduces the cell response to oxidative stress. Material 1 (crosslinked Vitamin E—stabilized UHMWPE) stimulated an increase in OGT/OGA ratio but in a minor extent of its not-crosslinked counterpart (material 3), indicating that the cross linking process contributes to generate oxidative stress and weakly reduces the beneficial effect of Vitamin E-stabilization. This is in accordance with the literature reporting that the cross linking process is a strong source of UHMWPE oxidation (6, 32, 33). Taken together, these results suggest that the Vitamin E-stabilization, in particular in absence of cross linking, stimulates cellular response to oxidative stress, in order to promote cell survival.

The results of ROS production are in line with recent literature evidences indicating that UHMWPE induce oxidative stress, even when modified (34) with Vitamin E. Indeed, Vitamin E exerts an antioxidant effect mainly *in vivo* as a scavenger of oxidant molecules, in particular lipid-soluble peroxyl radical, when they reach a high concentration, in order to prevent cell damage (35). On the other hand, is should be noted that low levels of oxidative stimuli, such as ROS generation (36), induce a cellular adaptive response to upregulate the defense capacity against subsequent oxidative stress (35, 37–39). Consistently, O-GlvNAcylation response proceeds as a two-step process. An initial ROS stimulus stimulates O-GlvNAcylation which, in turn, activates an intracellular signaling cascade leading to a long-term oxidative defense process. Indeed, a significative reduction in ROS generation is reported to be reduced by a sustained O-GlvNAcylation (40). In our results, the early and transient ROS production in response to M1 and M3 could be considered a priming factor that induces, at later time points, the O-GlvNAcylation as a mechanism of long-term antioxidant defense. Indeed, at a time point of 96 h, O-GlvNAcylation was observed in concordance with a decrease in ROS and a stabilization, with no significative difference between the three materials. These results indicate that Vitamin E added to WHMWPE does not have an immediate antioxidant effect (24 h) on the reduction ROS generation, but a more long term effect, stimulating, by an initial and transient ROS increase, the O-GlvNAcylation, which leads in turn to a antioxidant defense process.

These results are consistent with the final goal of Vitamin E adding to UHMWPE, to prevent the long-term effect of UHMWPE oxidation.

Oxidative stress is related to the inflammatory response, which in turn, affects bone turnover and remodeling by means of osteoimmunological mediators (9).

Vitamin E has also been shown to influence inflammatory cytokine production (17, 41). We recently showed the effect of Vitamin E—stabilized UHMWPE wear particles on osteoimmunological molecule's gene expression and secretion at early time points (26), so in this study, we aimed to analyze in parallel the effects of Vitamin E added to UHMWPE on oxidative stress response and osteoimmunological response. In particular, O-GlcNAcylation has been reported to affect the production of osteoimmunological factors (42). In our study, the osteoimmunological factors RANKL and its negative regulators confirmed our previous report, showing an increase in the bone-resorptive marker RANKL and a correspondent reduction of the osteo-protective marker OPG in response to UHMWPE without Vitamin E, while when vitamin E is added to stabilize UHMWPE, there is an strong reduction in RANKL, and an increase in OPG. In order to give a comprehensive result, we measured the RANKL/OPG ratio, which is considered a better parameter than the single RANKL and OPG values to evaluate the trend toward bone resorption or bone formation stimuli (43). A number of studies have shown that bone remodeling is dependent on the ratio of RANKL to OPG: if RANKL is higher bone resorption dominates, while when OPG is higher, the balance is shifted toward bone formation (44, 45). Thus, OPG acquired its name from its ability to protect bone from excessive resorption by counteracting the osteoclastic effects of RANKL (46, 47). Over the last decade, innovative, efficacious treatments for osteolysis have been developed specifically targeting the RANKL/OPG ratio, in order to reduce the incidence of related implant failures (43). In our study, RANKL/OPG ratio showed a trend toward an increase in this ratio when UHMWPE is not stabilized with Vitamin E, while the addition of Vitamin E restores the bone turnover stimuli to levels comparable to controls.

These results are also confirmed by the evaluation of two other main osteoimmunological factors, Sclerostin (SOST), and DKK-1, two inhibitors of the Wnt pathway involved in bone remodeling regulation. Wnt Signaling stimulates OPG expression, while the Wnt inhibitors Sclerostin and DKK-1 prevent this effect (13), thus promoting bone resorption. Our results confirmed an increase in Sclerostin expression in correspondence to a decrease in OPG expression, in response to material 2 (UHMWPE without Vitamin E), and a decrease of Sclerostin in correspondence to the treatment with Vitamin E-stabilized UHMWPE. In this case, no significant difference was observed among crosslinked and not-crosslinked Vitamin E-stabilized UHMWPE, suggesting that the cross linking process does not affect the expression of these osteoimmunological biomarkers. A different effect was observed for DKK-1 secretion, showing no significant decrease in response to material 1 and a little increase in response to material 3, while as expected it displayed an increase, even though not significant, in response to material 2. The exact mechanism of DKK-1 regulation of OPG production still needs to be elucidated, and these results suggest that it could be different from the Sclerostin mechanism. It is known that Sclerostin and DKK-1 can act separately and even alternatively in the regulation of bone turnover (48). We have already shown that Vitamin E-stabilized UHMWPE wear debris had different effects on Sclerostin and DKK-1 (26), and recent evidence indicated that these two Wnt inhibitors can be differently influenced by treatments (49) or pathological conditions (50). Consistently with these results, recent evidence showed that redox regulating mechanisms are able to affect cytokine and osteoimmunological factors produced by bone tissue and that the expression of RANKL, OPG and Sclerostin are redox regulated processes (51, 52). In particular, among Wnt pathway inhibitors, Sclerostin can be affected by oxidative stress, showing the same reduction in response to antioxidant as RANKL expression and RANKL/OPG ratio (52).

The limitation of this study is that the effect of Vitamin E stabilization was not tested on primary human osteoblasts, which could better reproduce the *in vivo* conditions, but on an immortalized human osteoblast cell line. Nevertheless, this choice was made in order to introduce as few variables as possible, since the effect of Vitamin E stabilization of UHMWPE on osteoblasts was unknown. Osteoblastic–like SaOS-2 cells are considered a valuable system for studying osteoblast functions and response to oxidative stress (10). Moreover, the choice for this immortalized cell line provides some advantages, such as a more stable and standardized growth condition than the primary cell culture.

In conclusion, taken together, these results suggest that the Vitamin E stabilization of UHMWPE produces two synergic effects on osteoblasts: on the one hand, it improves the ability of osteoblasts to respond to oxidative stress, inducing the cellular mechanism of defense, such as dynamic O-GlcNAcylation; on the other hand, the antioxidant effect influences the secretion on osteoimmunological factors, stimulating bone protective osteoimmunological factors such as OPG and reducing the RANKL/OPG ratio. This effect observed *in vitro*, could reflect *In vivo* through inducing a shift of the bone turnover balance toward bone protection. This suggests that the Vitamin E-Stabilization of UHMWPE could contribute to reduce oxidation- induced osteolysis (6, 32, 53) and the consequent loosening of the prosthetic device, therefore, improving the longevity of total joint replacements.

## Data Availability

The raw/processed data required to reproduce these findings cannot be shared at this time due to legal or ethical reasons

## Author Contributions

LM, EG, and VR contributed conception and design of the study. NP, LM, and GG performed the oxidative stress evaluation. MMCR and GG revised critically data interpretation. EG wrote the draft of the manuscript and performed osteoimmunological assays. All authors contributed to manuscript revision, read, and approved the submitted version.

### Conflict of Interest Statement

EG is the Principal investigator of a grant “Ricerca Comissionata” (#12973) of the University of Milan founded by Permedica S.p.A. VR is employed by Permedica S.p.A. The remaining authors declare that the research was conducted in the absence of any commercial or financial relationships that could be construed as a potential conflict of interest.
